# Completely mitochondrial genome of *Neolissochilus heterostomus*

**DOI:** 10.1080/23802359.2021.1945966

**Published:** 2021-08-19

**Authors:** Jinghong He, Chenfeng Zhao, Yongyao Guo, Haixia Zhang, Bo Zhao, Zhangjie Chu

**Affiliations:** Zhejiang Ocean University, Zhoushan, China

**Keywords:** Mitochondrial DNA, sequence analysis, *Neolissochilus heterostomus*

## Abstract

In this study, we determined the complete mitochondrial genome of *Neolissochilus heterostomus*. The genome is 16,585 bp in length, including 2 ribosomal RNA genes, 13 proteins-coding genes, 22 transfer RNA genes, and two non-coding control regions. Sequence analysis showed that the overall base composition of *N. heterostomus* is T 24.8%, C 27.7%, A 31.7%, and G 15.8%. The sequence is a slight A + T bias of 56.5%, which is similar to other fishes. We describe a phylogenetic analysis of 16 species of Cypriniformes based on the complete mitochondrial genome, and the result showed that *N. stracheyi* is most closely related to *N. heterostomus*. This mitogenome sequence data would play an important role in the investigation of phylogenetic relationship of the Cyprinidae.

*Neolissochilus heterostomus* is a endemic Cyprinidae fish species in China (Chen 2013). Currently a small amount is distributed in the Daying River Basins of Yunnan Province. The taxonomic status of *Neolissochilus* is still unclear. Therefore, it is very important to characterize the complete mitochondrial genome of this species, which can be utilized in research on taxonomic resolution, population genetic structure and phylogeography, and phylogenetic relationship.

In this study, we sequenced the complete mitogenome of *N. heterostomus*. The specimen was obtained from Daying River, Tengchong city, Yunnan province, China (24°36′36ʺN, 97°49′12ʺE). A tissue sample was collected from this specimen and deposited at the biological specimen room of Zhejiang Ocean University (www.zjou.edu.cn, Jinghong He, 2311537495@qq.com) under the voucher number LJL20201224. The total genomic DNA was extracted from tail muscle tissues by Phenol–chloroform extraction (Russell and Sambrook 2001). The complete mitochondrial genome sequences were amplified by 16 pairs of primers designed on the basis of related species mtDNA sequences by Primer Premier 5.0 (Supplementary Table S1).

The complete mitochondrial genome is 16,585 bp in length, including 2 ribosomal RNA genes, 13 protein-coding genes, 22 transfer RNA genes, and two non-coding control region. The overall base composition of *N. heterostomus* is T 24.8%, C 27.7%, A 31.7%, and G 15.8%. The sequence is a slight A + T bias of 56.5%. Most of the protein-coding genes used ATG as the initial codons (ND1, ND2, COX2, ATP8, ATP6, COX3, ND3, ND4L, ND4, ND5, ND6, Cytb), except for COX1 genes, which used GTG instead of ATG. Six protein-coding genes ended with the terminal codon, TAA (ND1, COX1, ATP6, ND4L, ND5, ND6), Gene ND2, ND3 and ATP8 used TAG as the terminal codon, and COX3 use TA instead of TAA. COX2, ND4 and Cytb shared the incomplete terminal codons T. Except for eight tRNA (tRNA^Ser^, tRNA^Pro^, tRNA^Glu^, tRNA^Tyr^, tRNA^Cys^, tRNA^As^, tRNA^Ala^, tRNA^Gln^) and the ND6 genes encoded on the L-strand, the other genes are encoded on the H-strand. The complete mitgenome sequence of *N. heterostomus* has 16SRNA (1637 bp) and 12SRNA (954 bp), which are located between tRNA^Phe^ with tRNA^Leu(UUR)^. Two non-coding regions are found in *N. heterostomus* mitogenome, the OH (913 bp) gene located between tRNA^Pro^ and tRNA^Phe^, and an OL (32 bp) located between tRNA^Asn^ and tRNA^Cys^. The 21 tRNA genes, ranging from 66 to 76 bp in size. There are two reading frame overlaps occur on the same strand: ATP8 and ATP6 overlap by 7 bp, and ND4L and ND4 overlap by 7 bp. The mitochondrial genome sequence of *N. heterostomus* was submitted to the GenBank (GenBank accession number: MW762597) and aligned with related sequences by BLAST.

The mitogenome sequence of *N. heterostomus* and allied species (Jiang 2019; Xu et al. 2019) was analyzed with the G + I + GTR model of the maximum likelihood (ML) method using MEGA version 7.0 software, and phylogenetic tree was constructed through bootstrap 2000 replicates. The 15 mitogenome sequences were downloaded from GenBank in NCBI, and *Gyrinocheilus aymonieri* was used as an out group for the phylogenetic analysis (Figure 1).

**Figure 1.**
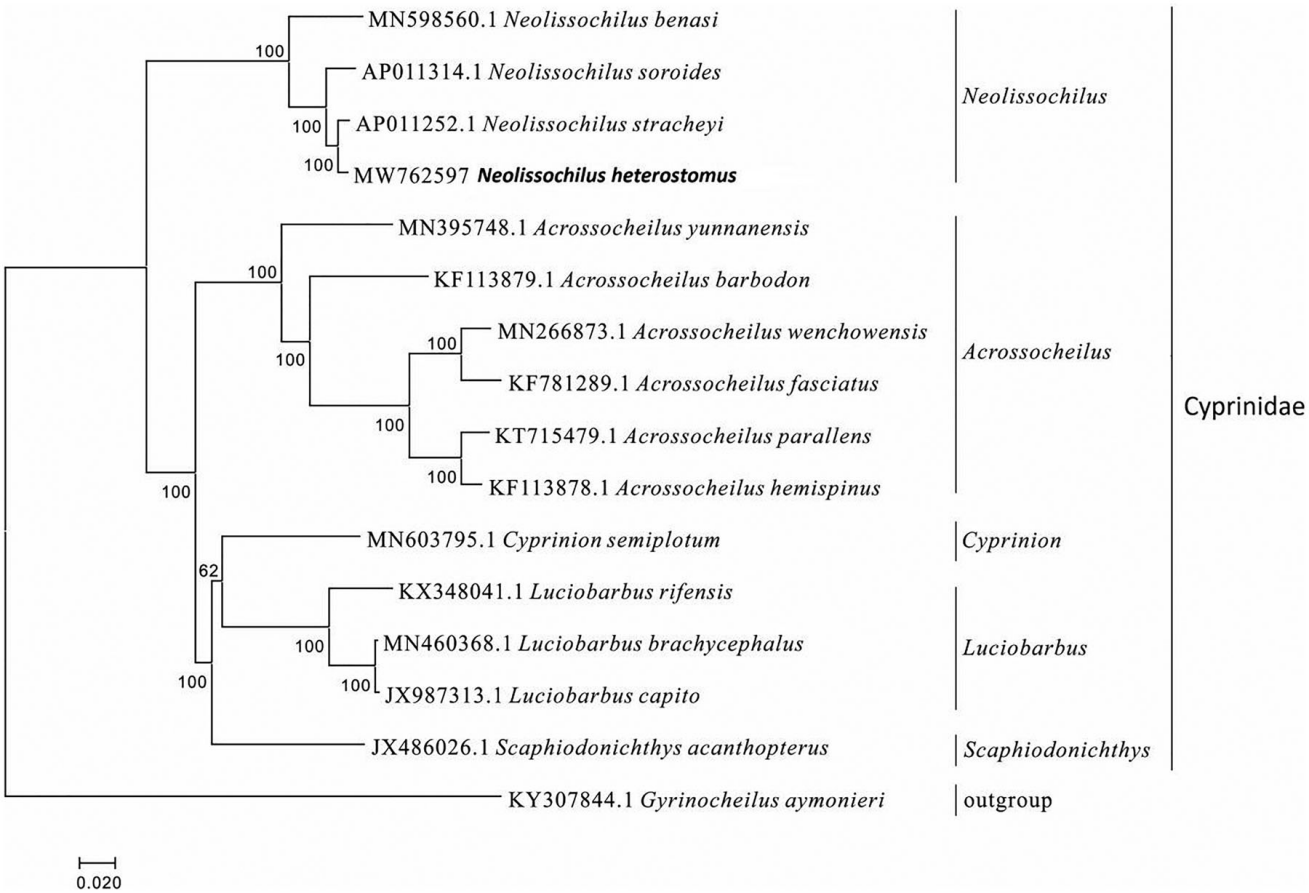
Phylogennetic analysis of *Neolissochilus heterostomus* based on the entire mtDNA genome sequences of 16 Cypriniformes available in GenBank. Numbers above the nodes indicate 2000 bootstrap values. Accession numbers are shown before species names.

The phylogenetic analysis showed that *N. stracheyi* was closely related to *N. heterostomus*, and *Neolissochilus* had closest relationship with *Acrossocheilus*. We expect that the present result will facilitate the further investigations of phylogenetic relationship, taxonomic resolution and phylogeography of the Cyprinidae.

## Data Availability

The genome sequence data that support the findings of this study are openly available in GenBank of NCBI at (https://www.ncbi.nlm.nih.gov/) under the accession no. MW762597. The associated BioProject, SRA, and Bio-Sample numbers are PRJNA732218, SRX10970856, and SAMN19314210/ SAMN19314211, respectively.
